# MiR-4733-5p promotes gallbladder carcinoma progression via directly targeting kruppel like factor 7

**DOI:** 10.1080/21655979.2022.2065951

**Published:** 2022-04-21

**Authors:** Xiaoqiang Hu, Junzhe Zhang, Junfeng Bu, Kaini Yang, Sunwang Xu, Mengqiao Pan, Dongxi Xiang, Wei Chen

**Affiliations:** a Department of Biliary and Pancreatic Surgery, Renji Hospital Affiliated to Shanghai Jiao Tong University School of Medicine, Shanghai 200120, China; b State Key Laboratory of Oncogenes and Related Genes, Shanghai Cancer Institute, Renji Hospital Affiliated to Shanghai Jiao Tong University School of Medicine, Shanghai 200120, China; c Shanghai Key Laboratory of Biliary Tract Disease, Renji Hospital Affiliated to Shanghai Jiao Tong University School of Medicine; Shanghai 200120, China; d Shanghai Research Center of Biliary Tract Disease, Renji Hospital Affiliated to Shanghai Jiao Tong University School of Medicine, Shanghai 200120, China

**Keywords:** Gallbladder cancer, MiRNA, epithelial-mesenchymal transition, MiR-4733-5p, KLF7

## Abstract

Gallbladder carcinoma (GBC) is highly aggressive with poor prognosis. Accumulating reports show that miRNAs play critical roles in tumor progression. Previous studies have identified several miRNAs that promoted or inhibited GBC cell proliferation and/or metastasis. Here, we used the Gene Expression Omnibus (GEO) dataset to identify dysregulated miRNAs in GBC, followed by validating the upregulation of the miR-4733-5p and downregulation of kruppel-like factor 7 (KLF7) in GBC biopsies by quantitative real-time PCR (RT-qPCR), in situ hybridization (ISH) staining, and immunohistochemistry (IHC) assays. GBC cell proliferation and invasion capacities mediated by miR-4733-5p were evaluated by a series of function assays *in vitro*, including CCK-8, colony formation assay, wound healing assay and transwell assay. Xenograft tumor model found that miR-4733-5p promoted GBC tumor growth *in vivo*. This study clarified that miR-4733-5p was upregulated in GBC and promoted GBC cell proliferation via directly binding to 3’ untranslated region (UTR) of *KLF*, which was downregulated and prohibited the proliferation and migration of GBC cells.

## Graphical abstract

**Figure:**
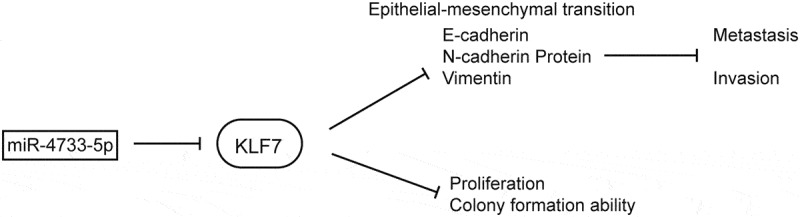

## Introduction

GBC is an uncommon malignancy with a low incidence in developed countries, but in endemic regions such as southern Chile (27/100,000), northern India (21.5/100,000), Poland (14/100,000) and Japan (7/100,000), GBC is still a substantial health problem [1]. GBC is highly aggressive with atypical symptoms; most patients are diagnosed at an advanced stage without opportunities for surgical resection [2]. Many studies have recently revealed the common mutated genes and their roles in GBC development [3–5], research on the mechanism underlying the initiation and progression of GBC is far from sufficient.

MiRNAs are noncoding RNAs with a length of approximately 22nt that act at the posttranscriptional level by binding to the 3’ UTR of target gene to inhibit mRNA translation [6]. According to the different types of target genes, miRNAs can be divided into oncogenic and tumor suppressive miRNAs [7]. Previous studies have found that miR-20a, miR-29c-5p, miR-30a-5p and miR-663a directly targeted Smad7, cytoplasmic polyadenylation element-binding protein 4 (CPEB4), E2F transcription factor 7 (E2F7) and epithelial membrane protein-3 (EMP3), respectively, and played important roles in GBC progression [8–11]. However, there are still several differentially expressed miRNAs in GBC, and their underlying mechanisms remain unclear. Here, we first analyzed differentially expressed miRNAs in GBC through a GEO dataset and discovered miR-4733-5p is upregulated in GBC tissues. However, the specifical biological role of miR-4733-5p in GBC is still unknown.

In this study, we focused on evaluating the function and potential mechanism of miR-4733-5p in facilitating GBC progression. We identified that miR-4733-5p worked as an oncogenic miRNA in GBC that promoted the proliferation, migration, and invasion of GBC cells, and epithelial-mesenchymal transition (EMT) is a potential driven mechanism induced by miR-4733-5p in GBC cells. The KLF7 was identified as a target gene of miR-4733-5p, which was downregulated in GBC and inhibited GBC cells proliferation and metastasis.

## Materials and methods

### Differential expression analysis of miRNAs

The miRNA microarray data were obtained from GEO database with accession number GSE104165 (GEO Accession viewer (nih.gov)). Data were normalized and analyzed using R package limma. Differentially expressed miRNAs (DEmiRNAs) were detected between GBC and normal gallbladder (NGB) tissues. Log FC ≥ 2 or ≤ −2 and *P < 0.05* was used as the thresholds to identify DEmiRNAs.

### Human tissue samples

Human GBC tissues and paired adjacent noncancerous gallbladder tissues (> 5 cm away from the GBC tissues) were retrospectively obtained from 28 patients who underwent surgical treatment and were diagnosed as GBC through histopathological examination by two pathologists during 2019–2020 at the Department of Biliary-Pancreatic Surgery, Ren ji Hospital, School of Medicine, Shanghai Jiao Tong University. Each patient included in this study provided informed written consent and their tissue samples were only used for scientific research. For miR-4733-5p and KLF7 expression level validation, total RNA was extracted with TRIzol reagent (Invitrogen, USA) from frozen tissue samples, fresh tissue samples were fixed using formalin and embedded with paraffin for in situ hybridization (ISH) staining assays and immunohistochemistry (IHC) assays.

This study was approved by the Research Ethics Committee of Ren ji Hospital, School of Medicine, Shanghai Jiao Tong University.

### Cell lines and cell culture

The human GBC cell lines (NOZ and GBC-SD) were purchased from the Shanghai Institute for Biological Science, Chinese Academy of Science (Shanghai, China). SGC-996, OCUG-1 and EHGB-1 cell lines were kind gifts from Prof. Yingbin Liu. All cell lines were cultured in high-glucose Dulbecco’s modified Eagle’s medium (DMEM) (Gibco, South American) supplemented with 10% fetal bovine serum (FBS) (Gibco, South American), and 1% penicillin and 1% streptomycin in a humidified incubator at 37°C with 5% Carbon Dioxide.

### Cell transfection and generation of stable cell lines

For knockdown or overexpression of miR-4733-5p, hsa-miR-4733-5p mimic and inhibitor and their corresponding negative control (NC) RNAs were purchased from Sangon Biotech (Shanghai, China). The sequences of RNA oligonucleotides used in this study were listed in Supplementary Table1. NOZ and GBC-SD cells were cultured to 30%–50% density and then transfected with RNA oligonucleotides using Lipofectamine 2000 (Invitrogen, California, USA). The detail methods were described previously [12]. The concentration of each RNA oligonucleotides used was 80 nM. After 48 hours, cells were harvested for subsequent experiments.

The open reading frame (ORF) sequences of human *KLF7* were cloned into the pLX304-V5-Blast vector and synthesized by GenePharma (Shanghai, China), the LV3-miR-NC and LV3-miR-4733-5p inhibitor Spong plasmids were also purchased from that company. The small hairpin RNAs (shRNAs) of *KLF7* plasmids pGIPZ-shKLF7-1, 2 and empty vector (EV) were synthesized and purchased from the DNA library of Shanghai Jiao Tong University School of Medicine (https://dnacore.shsmu.edu.cn). The sequence of shKLF7-1, shKLF7-2 and KLF7 were listed in Supplementary table2. Generation of stable cell lines were constructed according to the methods described previously [3].

### Cell growth and colony formation assays

For cell proliferation ability assay, NOZ or GBC-SD cells were calculated and seeded at 2 x 10^4 cells per well (100ul) in 96-well plates after transfected with RNA oligonucleotides. At suitable time points (24 h, 48 h, 72 h and 96 h post plating), the Cell Counting Kit-8 solution (Yeasen, Shanghai, China) was added into each well (10 ul per well), and 2 hours later, the absorbance at a wavelength of 450 nm was evaluated [13].

For colony formation assays, 1 x 10^3 NOZ cells and 2 x 10^3 GBC-SD cells were seeded in six-well plates. Culture medium with 10% FBS was refreshed every 2 days. After about 2 weeks, the plates were washed using Phosphate Buffered Saline (PBS) and stained with 1% Crystal Violet Staining Solution (Beyotime, Shanghai, China) and photographed. Colonies were counted and analyzed using the ImageJ software.

Each experiment was repeated independently three times.

### Wound healing and transwell assays

For wound healing assays, GBC-SD cells transfected with mimic or inhibitor were seeded into six-well plates, after 24 hours when cells reach confluence, a linear scratch ‘wound’ was created with a 10 μl pipette tip and then captured images at regular intervals by time-lapse microscope.

Migration and invasion ability assays were performed using 24-well plates with a chamber containing cell permeable membrane with or without Matrigel® matrix (Corning, NY, USA), 1 x 10^4 cells were added into the upper chamber with 200 ul serum-free DMEM culture medium, and 500 ul DMEM with 10% FBS was added into the lower chamber. The 24-well plates were incubated for 24 hours, and then cells were fixed using 4% paraformaldehyde and stained with 1% Crystal Violet Staining Solution, and cells on the upper membrane surface were gently removed and the lower were photographed. Cells were counted and analyzed using the ImageJ software.

Each experiment was repeated independently three times.

### RNA isolation and RT-qPCR

Total RNA was extracted from cells and tumor tissues using TRIzol reagent (Invitrogen, USA), and cDNA was reverse transcribed by PrimeScript RT regent Kit (Takara, Osaka, Japan). RT-qPCR assay was performed using ChamQTM Universal SYBR Green qPCR Master Mix (Vazyme Biotech co., Ltd, Nanjing, China) according to the manufacturer’s instructions. The relative gene expression was calculated using the comparative Ct method with ACTB or U6 as the control. Detail methods for RT-qPCR assay were described previously [14]. The primers used in this study were listed in Supplementary table 3. Each experiment was repeated independently three times.

### Western blotting

RIPA lysis buffer (Beyotime, China) added with freshly 1% PMSF (Beyotime, China) and 1% protease inhibitor cocktail (Yeasen, China) was used to extract the total proteins from cells and tissue samples. The total protein was quantified by BCA Protein Assay Kit (Thermo scientific, USA), and separated by sodium dodecyl sulfate-polyacrylamide gel electrophoresis (SDS-PAGE) and then transferred onto the polyvinylidene difluoride (PVDF) membranes. The PVDF membrane was then blocked with 5% nonfat milk for 1 hour, washed in TBS-T buffer for 15 min and then incubated with primary antibodies overnight at 4°C. After incubated with Horseradish Peroxidase-conjugated secondary antibodies, the immunoblot bands were detected with Lumi Q ECL reagent solution (Share-Bio, China). The primary and secondary antibodies were purchased as follows: KLF7 (1:1000, #sc-398,576, Santa Cruz, USA), E-cadherin (1:1000, #A3044, ABclonal, China), N-cadherin (1:1000, #A0432, ABclonal, China), Vimentin (1:1000, #A2584, ABclonal, China), GAPDH (1:1000, #5174, Cell Signaling Technology, USA) and β-Tubulin (1:1000, #AC030, ABclonal, China). Each experiment was repeated independently three times.

### Dual luciferase reporter assay

The 3’UTR of KLF7 containing the predicted miR-4733-5p binding site was amplified by PCR from H293T genomic DNA and subcloned into a pGV272-control reporter vector (GeneChem. Shanghai, China) to construct the KLF7-wild type (WT). The mutant 3’-UTR of KLF7, which contained point-mutated sequence in the binding region of miR-4733-5p, was generated using a site-directed mutagenesis kit (TransGen Biotech, Beijing, China). Reporter plasmids (4.0 nmol) and pGV272-control (500 ng) were transfected into H293T cells with Lipofectamine 2000 (Invitrogen), and 4 nM of NC-mimic or miR-4733-5p mimic was transfected into H293T cells. Cells were collected after 48 h for analyzed using dual-luciferase reporter assay kit (Vazyme Biotech co., Ltd, Nanjing, China). Each experiment was repeated independently three times.

### Xenograft tumor model

For animal experiments, 5- week-old male BALB/c nude mice were purchased from SLAC Laboratory Animal Co., Ltd. (Shanghai, China). They were bred in laminar flow cabinets under specific pathogen-free conditions. NOZ cells (1x10^6) stably expressing LV3-miR-NC or LV3-miR-4733-5p inhibitor sponge with 100ul PBS were subcutaneously injected into the right axilla of each mouse (n = 5 mice per group) to establish the xenograft models. The length and width of the tumors were measured with calipers every week after injection. All mice were killed after 4 weeks, and subcutaneous tumors were isolated and weighed. The volume of the tumor was calculated by using the formula: volume = (width^2^ x length)/2. The animal assays were approved by the Animal Ethics Committee of Ren ji Hospital, School of medicine, Shanghai Jiao Tong University.

### In situ hybridization (ISH) staining

Digoxigenin-labeled miR-4733-5p probe was used to detect the miR-4733-5p expression in GBC and adjacent NGB tissues. The sequence of miR-4733-5p probe was as follows: 5’-CACCGGGUCUAGCAUUGGGAUU-3’. The slides were de-paraffined and rehydrated before incubating with Proteinase K (20ug/ml) at 37°C for 20–30 min, then washed the slides three times with PBS (PH 7.4) for 5 minutes each time. After incubating with 5x SSC solution at room temperature for 15 min, the slides were added with miR-4733-5p probes for hybridizing overnight in the incubator at 37°C. Bovine Serum Albumin was added into the slides after the hybrid solution was washed using SSC buffer. And then the slides were incubated with anti-mouse-digoxin-labeled peroxidase. At last, 3,3’-diaminoben was used to immerse the tissue sections and the results of ISH were photographed and analyzed [15].

### Immunohistochemistry assay

Human and animal tissue samples were fixed in 10% formalin and embedded them in paraffin. Then we stained tissue sections with hematoxylin-eosin staining and accordingly antibodies to detect the KLF7 and Ki-67 protein expression levels. Detail methods for IHC assays were performed according to previously described protocols [12].

### Statistical analysis

SPSS (version 23.0, SPSS Inc.) and GraphPad Prism (version 7.0, USA) were used to analyze the experiment results. Medians and ranges of continuous variables were compared using the Student’ *t* test. Categorical variables were compared using the Pearson χ 2 test or Fisher exact test, as appropriate. All of the images of the migration assays, Western blot assays, Colony formation assays, ISH and IHC assays were representative of at least three independent staining or experiments results. The experiments of RT-qPCR, dual-luciferase reporter and proliferation ability were measured in at least triplicate, and each assay was repeated more than three times. *P* values were considered significant when less than 0.05.

## Results

Accumulating reports show that numerous miRNAs are abnormally expressed in tumor cells [16,17]. As an important regulator in the posttranscriptional stage of gene expression, miRNAs play critical roles in cancer progression [18,19]. This study focused on evaluating the role of miR-4733-5p in GBC, we performed a series of functional and molecular assays including CCK-8, colony formation assay, wound healing assay and transwell assay. In the end, we concluded that the upregulation of miR-4733-5p promoted GBC cell proliferation both *in vitro* and *in vivo*, enhancing GBC cell migration and invasion by activating the EMT process. KLF7 might be a direct target gene of miR-4733-5p, and KLF7 prevented GBC cell proliferation and migration.

## MiR-4733-5p expression level is elevated in human GBC tissues

**Figure 1.**
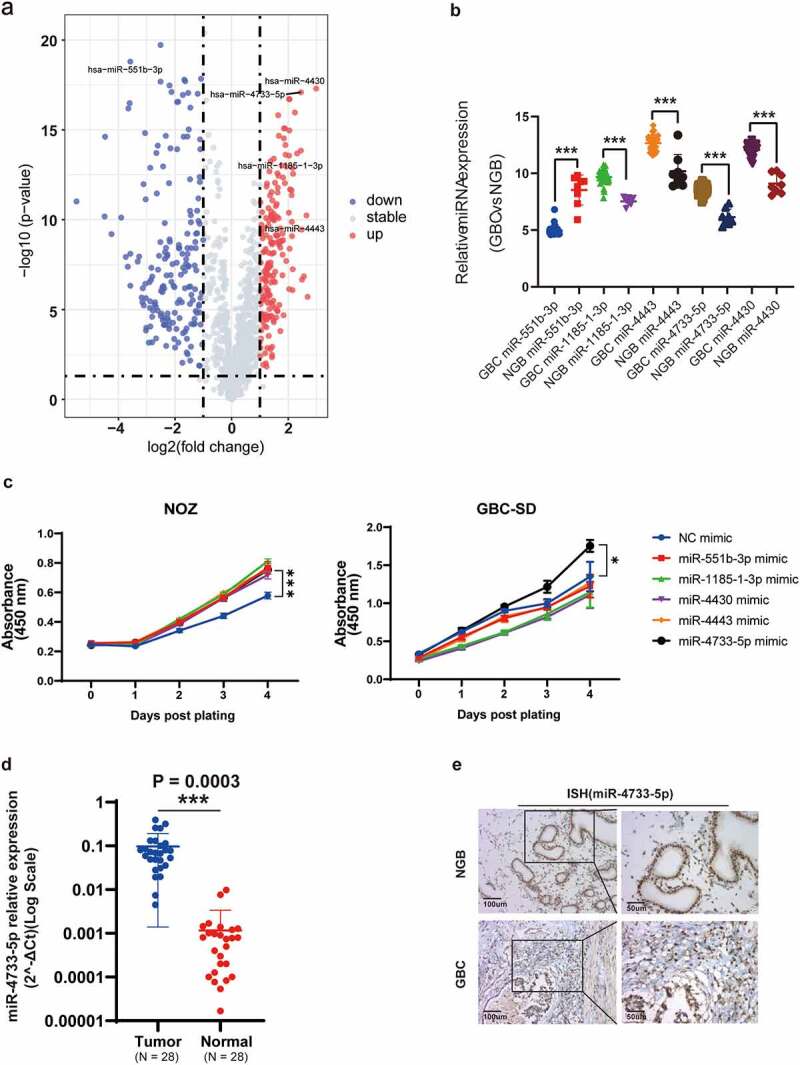
**miR-4733-5p is upregulated in GBC**. (a) Volcano plot of miRNA expression array from GSE104165 dataset. (b) The five differentially expressed miRNAs in GBC from GSE104165 dataset. (c) CCK-8 assays of NOZ and GBC-SD cells transiently transfected with five miRNA mimic and negative control mimic (NC-mimic). (d) Validation of miR-4733-5p expression in 28 pairs of GBC and adjacent normal tissues. (e) Representative ISH images of miR-4733-5p stained in 28 pairs of GBC and adjacent normal tissues by anti-miR-4733-5p probe, scare bar = 100 μm (left) and 50 μm (right). Unpaired Student’ s t test was used in **b, c** and **d** (*p <0.05, **p <0.01, ***p <0.001).

Analysis of the GSE104165 microarray dataset revealed 26 upregulated and 92 downregulated miRNAs in GBC compared with NGB tissues (log FC ≥ 2 or ≤ −2 and *P < 0.05*) (Figure 1a, Supplementary figure 1). Among these miRNAs, five differentially expressed miRNAs (miR-551b-3p, miR-1185-1-3p, miR-4443, miR-4733-5p or miR-4430) were selected for further study (Figure 1b, Supplementary table 4). We performed CCK-8 assay to examine the proliferative ability after overexpression of above-selected miRNAs in NOZ and GBC-SD cells. The results showed that overexpression of miR-4733-5p significantly promoted the proliferation of both NOZ and GBC-SD cells, while the rest of miRNAs showed no obvious differences in GBC-SD cells between the overexpression and NC groups (Figure 1c). We thus focused on miR-4733-5p for the subsequent investigation. We examined the expression level of miR-4733-5p in 28 pairs of GBC and NGB tissues by RT–qPCR and found a similar expression pattern (*N = 28, P = 0.0003*; Figure 1d). To confirm the results above, digoxigenin-labeled miR-4733-5p probes were synthesized to detect the miR-4733-5p expression, we found miR-4733-5p expression was higher in GBC tissues (Figure 1e). Taken together, these results indicate that miR-4733-5p is upregulated in GBC tissues.

## MiR-4733-5p promotes GBC cell proliferation and colony formation in vitro

**Figure 2.**
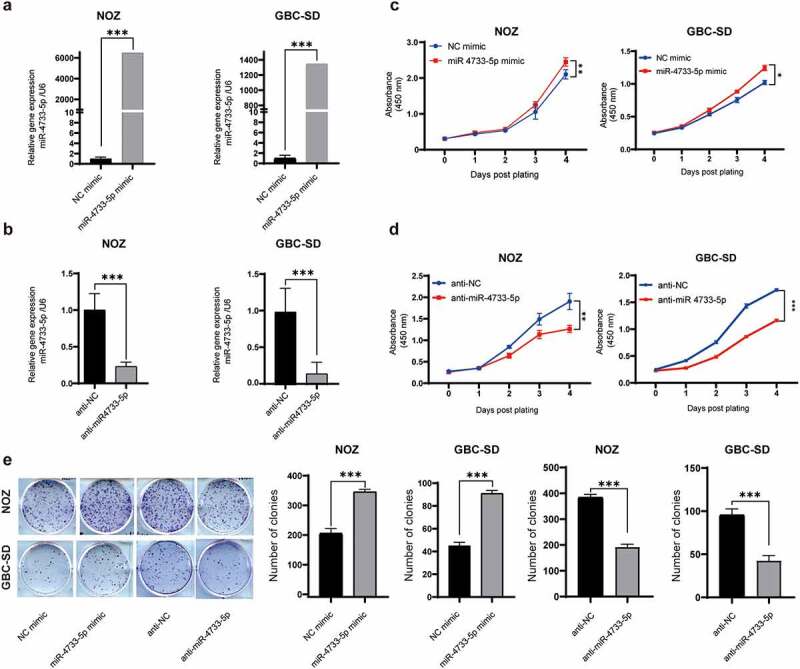
**miR-4733-5p promotes GBC cell proliferation and colony formation in vitro**. (a, b) The efficiency of miR-4733-5p overexpression (a) or knockdown (b) in both NOZ and GBC-SD cells transfected with miR-4733-5p mimic or miR-4733-5p inhibitor. (c, d) CCK-8 assay of NOZ and GBC-SD cells transfected with miR-4733-5p mimic or inhibitor and the corresponding NC mimic or anti-NC. (e) Colony formation assay of NOZ and GBC-SD cells transfected with the miR-4733-5p mimic or inhibitor and the corresponding NC mimic or anti-NC, representative images of colony formation assay (left), the number of colonies in each group was counted in the diagrams (right). Unpaired Student’ s t test was used in **a, b, c and d** (*p <0.05, **p <0.01, ***p <0.001).

To investigate the potential biological role of miR-4733-5p in GBC, we first examined the miR-4733-5p expression in five GBC cell lines (NOZ, GBC-SD, EHGB-1, SGC-996 and OCUG1). The results showed that the miR-4733-5p expression was highest in NOZ cells and lowest in GBC-SD cells (Supplementary figure 2). We overexpressed or knocked down miR-4733-5p expression in NOZ and GBC-SD cells with miR-4733-5p mimic or inhibitor, respectively, and the efficiency was confirmed by RT–qPCR (Figure 2a, b). The results of CCK-8 assays demonstrated that overexpression of miR-4733-5p increased NOZ and GBC-SD cell proliferation (Figure 2c), while knockdown of miR-4733-5p dramatically inhibited the proliferation of GBC cells (Figure 2d). Consistent with the CCK-8 assay results, colony formation assays showed that upregulation of miR-4733-5p expression significantly promoted the colony formation of GBC cells, whereas downregulation of miR-4733-5p suppressed this activity (Figure 2e). These data suggest that miR-4733-5p increases GBC cell proliferation and colony formation abilities.

## MiR-4733-5p promotes GBC cell migration and invasion via enhancing EMT process

**Figure 3.**
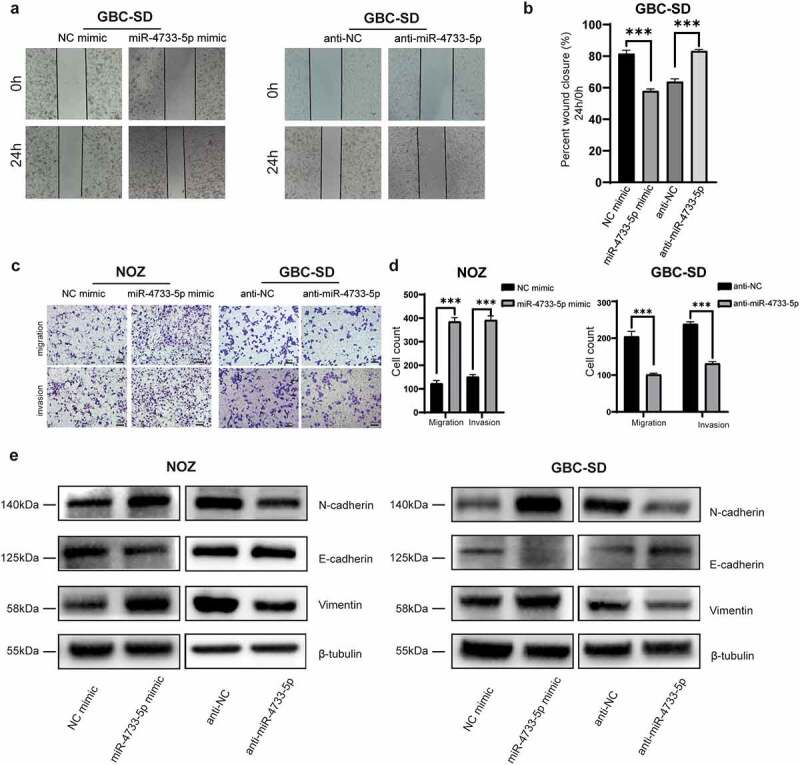
**miR-4733-5p enhances GBC cell migration and invasion by EMT process**. (a, b) Wound healing assay of GBC-SD cells transfected with miR-4733-5p mimic or inhibitor and the corresponding NC mimic or anti-NC. Representative images of colony formation assay (a), average percentage of closure of the scratch area was analyzed (b). (c, d) Invasion and migration (without Matrigel® matrix) assays of NOZ and GBC-SD cells transfected with miR-4733-5p mimic or inhibitor and the corresponding NC mimic or anti-NC. Representative images of colony formation assay, scale bar = 10 μm (c) and the cell count in each group was calculated in diagrams (d). (e) EMT-related protein (E-cadherin, N-cadherin and Vimentin) were examined by western blotting. Unpaired Student’ s t test was used in **b and d** (*p <0.05, **p <0.01, ***p <0.001).

To explore whether miR-4733-5p influences the migration of GBC cells in vitro, we first performed a wound healing assay with GBC-SD cells. The results revealed that overexpression of miR-4733-5p was correlated with notably faster wound closure, whereas downregulation of miR-4733-5p resulted in a slower rate (Figure 3a, b). We next performed the migration and invasion assay to validate the results of wound healing assay. Consistent with the results above, the migratory and invasive abilities of both NOZ and GBC-SD cells were significantly enhanced in the presence of miR-4733-5p but remarkably reduced in the anti-miR-4733-5p group (Figure 3c, d). The EMT refers to the loss of epithelial properties with gain of mesenchymal characteristics for better migration and proliferation [20–22]. To explore whether the EMT process is a mechanism by which miR-4733-5p depends on to promote GBC cell migration and invasion, EMT-related proteins (E-cadherin, N-cadherin and Vimentin) were examined in both NOZ and GBC-SD cells after transfected with miRNA mimic or inhibitor. The western blotting assay confirmed that in the miR-4733-5p mimic group, the expression of E-cadherin was remarkably decreased and N-cadherin and Vimentin was increased compared with the miR-NC group for both cell types. In contrast, cells treated with the anti-miR-4733-5p showed an opposite outcome (Figure 3e). Together, these results indicate that miR-4733-5p promotes the metastasis and invasion of GBC cells in vitro, at least partially by enhancing EMT process.

## *KLF7* is a direct target of miR-4733-5p

**Figure 4.**
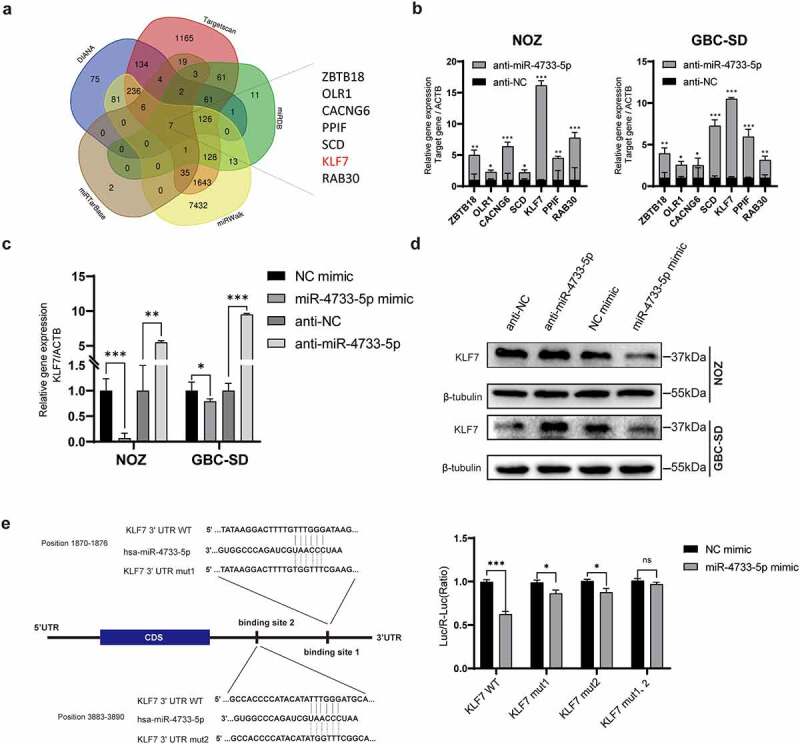
**miR-4733-5p directly targets the 3’ UTR of KLF7 and downregulates its expression**. (a) Veen diagram of possible target genes of miR-4733-5p predicted by five databases. The seven genes overlapped among these databases are listed in the right. (b) The RNA expression of seven predicted genes were examined in NOZ and GBC-SD cells transfected with miR-4733-5p inhibitor or anti-NC by RT–qPCR. (c, d) The RNA (c) and protein (d) expression of KLF7 was examined in NOZ and GBC-SD cells transfected with miR-4733-5p mimic or inhibitor and NC mimic or anti-NC by RT–qPCR and western blotting assay. (e) The relative luciferase activity was analyzed after co-transfected with KLF7 reporter plasmids and miR-4733-5p mimic into 293 T cells. The sequence of the potential miR-4733-5p binding sites in the 3’ UTR of KLF7 is shown in the left. Unpaired Student’ s t test was used in **b, c, and e** (ns: non-significant, *p <0.05, **p <0.01, ***p <0.001).

To explore the mechanisms by which miR-4733-5p induces GBC cell proliferation and migration, we searched five online databases for miRNA target prediction that might be potential targets of miR-4733-5p. Seven genes including *Zinc Finger and BTB Domain Containing 18 (ZBTB18), Oxidized Low Density Lipoprotein Receptor 1 (OLR1), Calcium Voltage-Gated Channel Auxiliary Subunit Gamma 6 (CACNG6), Peptidylprolyl Isomerase F (PPIF), Stearoyl-CoA Desaturase (SCD), KLF7* and *RAB30 (a member of RAS oncogene family)* overlapped among the five databases (Figure 4a). Compared to the anti-NC group, the anti-miR-4733-5p group showed remarkably increased expression of *KLF7* among them while overexpression of miR-4733-5p came to an opposite direction (Figure 4b-c). This observation was validation by western blotting assay (Figure 4d). To further confirm whether the KLF7 is directly regulated by miR-4733-5p, we constructed a dual-luciferase reporter plasmid containing a fragment of the *KLF7* 3’ UTR along with the miR-4733-5p putative binding sites. Co-transfection of miR-4733-5p mimic and the KLF7 3’ UTR expression vector into 293 T cells caused a remarkable repression of KLF7 luciferase activity. The luciferase activity of a single-mutant 3’ UTR of KLF7 was inhibited, both putative binding site 1 and 2 was eliminated in the mutated 3’ UTR of KLF7. When the two putative binding sites were both mutated, the luciferase activity was no difference between miR-NC group and miR-4733-5p mimic group (Figure 4e). Together, these data suggest that *KLF7* is a direct target gene of miR-4733-5p.

## MiR-4733-5p promotes GBC cell proliferation in vivo

**Figure 5.**
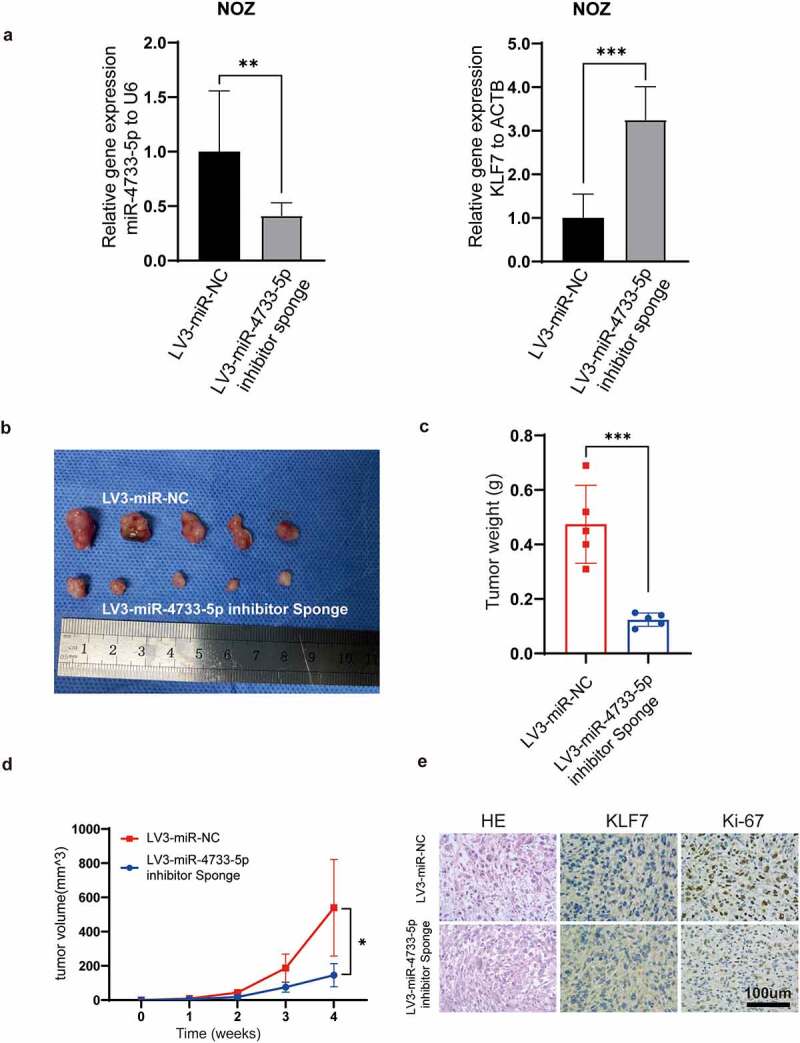
**miR-4733-5p promotes GBC tumor growth in vivo**. (a) The expression of miR-4733-5p (left) and KLF7 (right) in NOZ cells after stably transfected with LV3-miR-NC or LV3-miR-4733-5p inhibitor sponge. (b) Images of subcutaneous tumor in nude mice. (c, d) The tumor weight (c) and growth curve (d) of the xenograft tumors in each group. (e) Representative images of tumor slides were stained with hematoxylin and eosin or anti-KLF7 and anti-Ki-67 antibodies for IHC. Scale bar = 100 μm. Unpaired Student’ s t test was used in **a, c and d** (*p <0.05, **p <0.01, ***p <0.001).

To assess whether miR-4733-5p promotes the GBC growth in vivo, the stably transfected cell lines, LV3-miR-NC-NOZ and LV3-miR-4733-5p-inhibitor sponge-NOZ cell lines, were used to establish subcutaneous xenograft tumor models. KLF7 expression and the efficiency of miR-4733-5p knockdown were confirmed by RT–qPCR (Figure 5a). Tumor sizes and weights were significantly decreased in the LV3-miR-4733-5p-inhibitor sponge group compared to the control group (Figure 5b-d), which indicated that knockdown of miR-4733-5p inhibited GBC tumor growth. The expression of KLF7 in LV3-miR-4733-5p-inhibitor sponge group was increased while that of Ki-67 was decreased (Figure 5e). Taken together, our results indicate that miR-4733-5p can promote GBC growth in vivo.

## KLF7 restrains the malignant behavior of GBC cells

**Figure 6.**
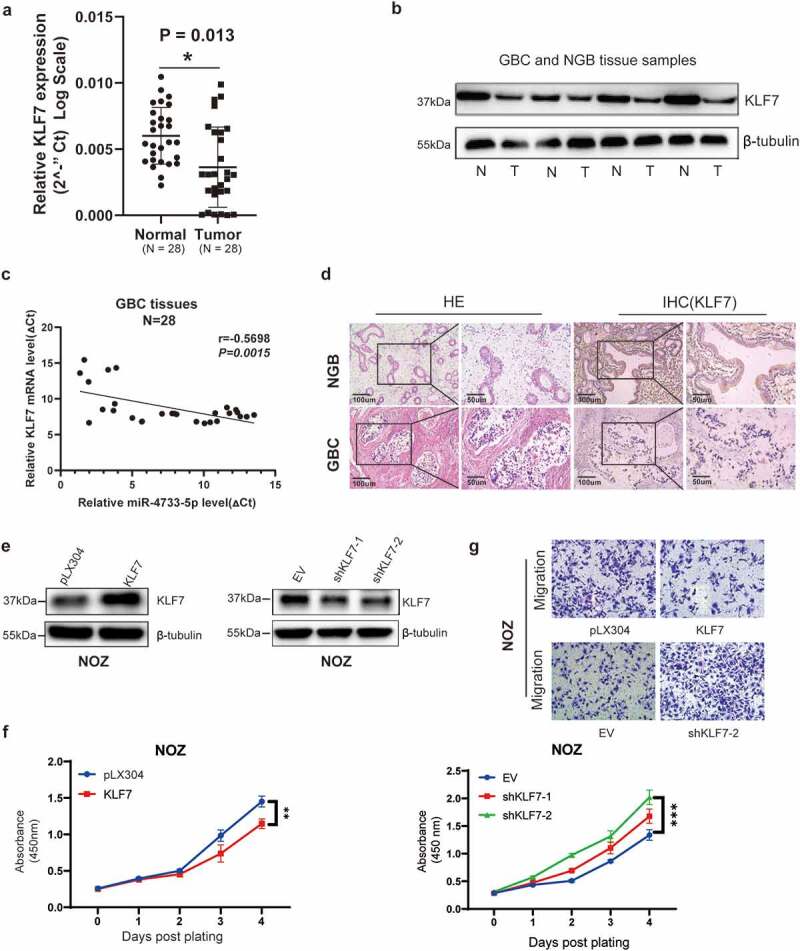
**Inhibition of KLF7 in GBC enhances cell proliferation and migration**. (a, b) The expression of KLF7 in 28 paired GBC and adjacent normal tissues by RT–qPCR (a) and western blotting (b). Representative images of western blotting are present (**b**). (c) The correlation between the expression of miR-4733-5p and KLF7 was analyzed using linear regression analysis (*P = *0.0015, *R = −0*.5698; Pearson’s correlation). (d) Representative images of KLF7 expression in GBC and NGB tissues by IHC staining. Scare bar = 100 μm (left) and 50 μm (right). (e) The KLF7 expression in NOZ cells were examined by western blotting after overexpression (left) or downregulation (right) of KLF7. (f, g) Cell proliferation (f) and migration (g) abilities were evaluated in NOZ cells after overexpression or downregulation of KLF7, scare bar = 10 μm. Unpaired Student’ s t test was used in **a, c and f** (*p <0.05, **p <0.01, ***p <0.001).

Given the fact that *KLF7* was a direct target gene of miR-4733-5p and the oncogenic role of miR-4733-5p in GBC, we hypothesized that *KLF7* might play a tumor suppressive role in GBC. We examined the RNA and protein expression levels of KLF7 in 28 GBC tissues and adjacent NGB tissues by RT–qPCR and western blotting assays, the results indicated that compared with NGB tissues, both the mRNA and protein expression levels of KLF7 were downregulated in GBC tissues (Figure 6a, b). The Pearson correlation analysis showed that miR-4733-5p expression was inversely correlated with *KLF7* expression in GBC samples (*R =* −0.5698, *P = *0.0015; Figure 6c). IHC staining verified the lower expression of KLF7 in GBC tissues (Figure 6d). We next evaluated the efficiency of downregulation or overexpression of KLF7 in NOZ cells transfected with shKLF7 plasmids or KLF7 expression plasmids, respectively (Figure 6e). Compared to the control groups, overexpression of KLF7 in NOZ cells attenuated proliferation ability, while KLF7 inhibition exhibited enhanced its proliferation (Figure 6f). As shown in Figure 6g, upregulation of KLF7 inhibited the migratory of NOZ cells. Collectively, these results indicate that KLF7 is downregulated in GBC tissues and it inhibits GBC cell proliferation and migration when KLF7 is overexpressed.

## Knockdown of KLF7 abrogated the inhibition of anti-miR-4733-5p on GBC cells

**Figure 7.**
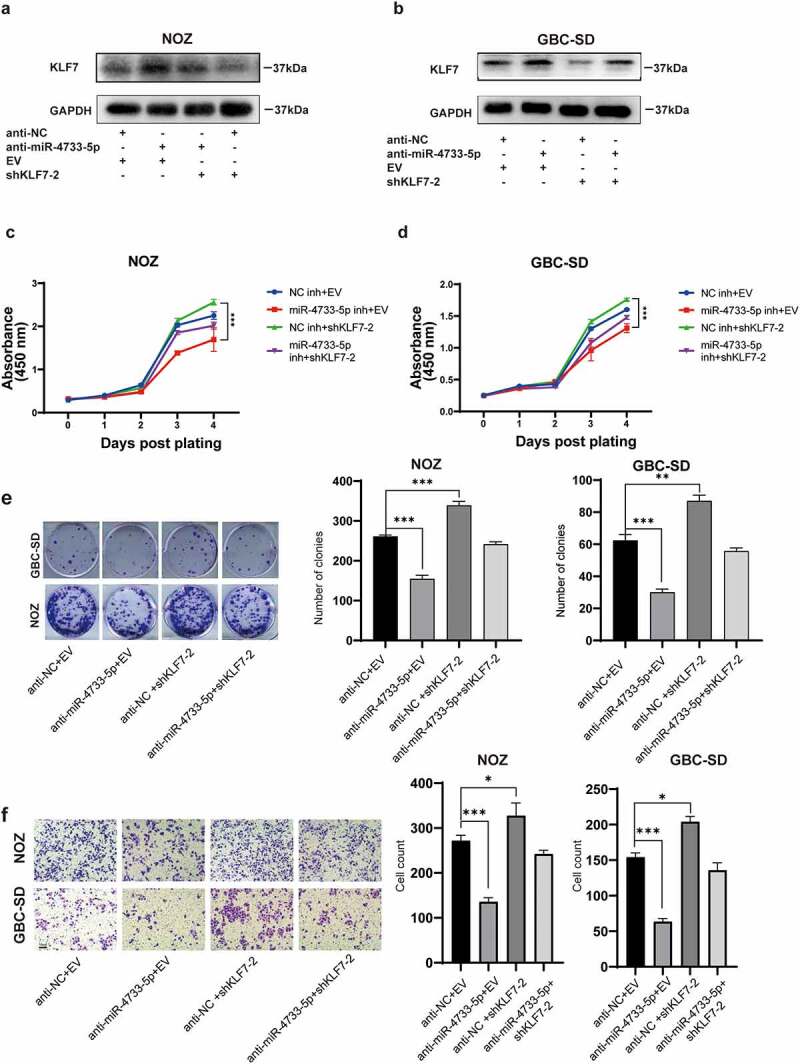
**Knockdown of KLF7 abrogated the inhibition of anti-miR-4733-5p on GBC cells**. (a, b) The KLF7 expression of NOZ and GBC-SD cells after transfected with anti-miR-4733-5p and shKLF7-2 plasmids. (c, d) Cell growth of NOZ and GBC-SD cells after co-transfection of anti-miR-4733-5p and shKLF7-2 plasmids. (e) Colony formation assays of NOZ and GBC-SD cells after co-transfection of anti-miR-4733-5p and shKLF7-2 plasmids. representative images of colony formation assay (left), the number of colonies in each group was counted in the diagrams (right). (f) Migration ability of NOZ and GBC-SD cells after co-transfection of anti-miR-4733-5p and shKLF7-2 plasmids. Representative images of migration assays were present (left) and cell count was calculated and analyzed (right). Unpaired Student’ s t test was used in **c, d, e and f** (*p <0.05, **p <0.01, ***p <0.001).

To determine whether the effects of miR-4733-5p in GBC progression were mediated by KLF7, we co-transfected anti-miR-4733-5p with shKLF7-2 plasmids into NOZ and GBC-SD cells. The protein level of KLF7 was upregulated by anti-miR-4733-5p in these cells, whereas KLF7 downregulation attenuated this alteration. Co-transfection of anti-miR-4733-5p and shKLF7 in GBC cells showed that anti-miR-4733-5p partially increased the downregulation of KLF7 expression caused by shKLF7 (Figure 7a, b). Cell proliferation was enhanced by KLF7 downregulation both in NOZ and GBC-SD cells, whereas the ability was reversed after GBC cells were co-transfected with anti-miR-4733-5p (Figure 7c, d). The colony formation ability was restricted by anti-miR-4733-5p, while co-transfection regained the colony number both in NOZ and GBC-SD cells (Figure 7e). NOZ and GBC-SD cells transfected with shKLF7 exhibited aggressiveness, while the co-transfection system partially abrogated the migration ability (Figure 7f). Taken together, these results interpret the role and regulatory mechanism of miR-4733-5p/KLF7 axis in GBC progression.

## Discussion

GBC is an extremely aggressive cancer with 5-year survival rate no more than 5%, especially for those advanced-stage patients. There still no curable treatment methods for advanced GBC patients [23,24]. It is critical to clarify the underlying mechanisms of GBC progression. miRNAs are reported as crucial regulators of posttranscriptional levels of genes, and these regulators play important role in the development and progression of cancers, including GBC [25,26]. It is meaningful to find the dysregulated miRNAs and elucidate their function, underlying mechanisms in GBC.

Cancer-related miRNAs in GBC have been substantially reported. Recent studies have identified extensively oncogenic and tumor suppressive miRNAs in GBC [11,27]. Numerous dysregulated genes were regulated by a series of cancer-related miRNAs and involved in cancer development and progression [28–30]. These cancer-related miRNAs tend to directly bind with 3’UTR region of target genes, inhibit the translation or accelerate the degradation process of message RNAs of target genes [7]. Upregulation of MiR-20a was closely related with local invasion, metastasis and worse prognosis of GBC patients, miR-20a promoted GBC cells metastasis by inducing the EMT process. Smad7, a famous oncogene, was identified as the target gene of miR-20a [8]. Shu et al [9] found that miR-29c-5p was downregulated in GBC tissues, ectopic expression of miR-29c-5p in GBC cells remarkably inhibited cell proliferation, migration, invasion and increased cell apoptosis rate. CPEB4 was confirmed as a direct target of miR-29c-5p. In this study, we identified the expression of miR-4733-5p was significantly upregulated in GBC. However, the precise role of miR-4733-5p in GBC progression was still largely unclear. CCK-8 and colony formation assays showed that cell proliferation and colony formation abilities were dramatically enhanced by overexpression of miR-4733-5p, tumor growth was remarkably repressed in the presence of anti-miR-4733-5p in vivo, these finding suggested miR-4733-5p has significant roles in cell proliferation and tumor growth.

Local invasion and distant metastasis are the main reasons for cancer-related death [31]. Aggressive migration capability is an important biological characteristic of cancer cells [32]. It is a complex process for cancer cells migrating from primary regions to distant organs [33,34]. EMT is a critical process that enables epithelial cells to acquire some mesenchymal traits and lose partially epithelial features [35]. Accumulating evidence shows that EMT-related gene mutation, such as *CDH1* and *Snail* gene, was discovered in various types of metastasis tumors [36,37]. The aberrant expression of these EMT-related genes is largely regulated by epigenetic modification [21,38,39]. As a critical regulator in posttranscriptional level, EMT-related genes also are modified by kinds of miRNAs. As the essential transcriptional repressors, Zinc Finger E-Box Binding Homeobox 1 (ZEB1) and ZEB2 were cooperatively regulated by miR-200 family and miR-205, downregulation of miR-200 family and miR-205 in breast cancer cells lost their ability to repress the ZEB1 and ZEB2 expression, thus the expression of E-cadherin was downregulated, which promoted tumor metastasis [40]. The mutation of CDH1 gene plays a vital role in the development of hereditary diffuse gastric cancer [36]. In this study, we found that ectopic overexpression of miR-4733-5p dramatically elevated the N-cadherin and Vimentin expression, while reduced the E-cadherin expression in GBC cells, suggesting that EMT process is a critical mechanism by which miR-4733-5p promotes GBC metastasis.

Furthermore, KLF7 was identified as a target gene of miR-4733-5p in this study. KLF7 was aberrant expression in various types of cancer. Romi Gupta et al [41] reported that KLF7 is upregulated in pancreatic cancer partially due to the inactivation of P53 gene, overexpression of KLF7 promotes tumor growth and metastasis by upregulating the expression of IFN-stimulated gene (ISG) and maintaining the integrity of Golgi complex. Interestingly, miR-103 was found upregulated in non‑small cell lung cancer and promoted cancer progression by directly targeting KLF7 [42]. Recent studies have also shown that KLF7 was downregulated in hepatocellular carcinoma (HCC), circUBE2J2 acted as a competing endogenous RNA to control KLF7 expression by sponging miR-370-5p. circUBE2J2/miR-370-5p/KLF7 ax plays a vital role in HCC development [43]. In the present study, KLF7 expression was also downregulated in GBC tissues. The GBC cell proliferation and migration abilities were remarkably inhibited by ectopic expression of KLF7, while KLF7 inhibition dramatically enhanced cell proliferation and migration abilities. Our results suggest that KLF7 acts as a tumor suppressor in GBC and it is partially regulated by miR-4733-5p.

Emerging evidence suggest that a miRNA can regulate various target genes and a gene can be regulated by different types of miRNAs [44,45]. Numerous types of potential genes were predicted by five target gene prediction databases in this study. Subsequently, seven genes were overlapped among the five databases and then KLF7 was identified as the potential target of miR-4733-5p. However, it is inevitable to lose many possible target genes that are also regulated by miR-4733-5p through such a target gene screening process. More accurate target gene prediction schemes, such as combination of predictive databases and next-generation sequencing technology, are needed to screen the target genes regulated by miR-4733-5p. In this work, we also identified that KLF7 was downregulated in GBC tissues, and overexpression of KLF7 in GBC cells dramatically inhibited cell proliferation and migration abilities. It was confirmed that KLF7 was regulated by miR-4733-5p in GBC. However, KLF7 may also be regulated by other important miRNAs in GBC, and multiple miRNAs including miR-4733-5p co-regulates the expression of KLF7, which needs further research. In this study, we evaluated the expression levels of EMT-related proteins and concluded that EMT process is a mechanism by which miR-4733-5p promotes GBC cell migration and invasion. However, the process and mechanism of tumor metastasis are quite complex [46,47], further deeply research should be designed to investigate other potential mechanisms by which miR-4733-5p promotes GBC metastasis. The mechanism of miRNA regulating tumor development and progression is complex, and this study needs to further explore whether miR-4733-5p affects the related signaling pathways in GBC.

## Conclusion

Taken together, this study identified that miR-4733-5p was upregulated in GBC tissues and promoted GBC cell proliferation, colony formation, migration and invasion, KLF7 was downregulated in GBC tissues and it might be directly regulated by miR-4733-5p. Additionally, miR-4733-5p enhanced the migration and invasion of GBC cell by promoting the EMT process. miR-4733-5p and KLF7 may be potential therapeutic targets for GBC therapy.

## Supplementary Material

Supplemental Material

## Data Availability

The miRNA microarray data were obtained from GEO database with accession number GSE104165 (https://www.ncbi.nlm.nih.gov/geo/). All data are available from the corresponding author on reasonable request.
