# Supplementary material for: MiR-4733-5p promotes gallbladder carcinoma progression via directly targeting kruppel like factor 7
Source: Bioengineered. 2022 Apr 21;13(4):10691–706. doi: 10.1080/21655979.2022.2065951 (PMC9161844; doi:10.1080/21655979.2022.2065951)
Supplement: Supplemental Material [file KBIE_A_2065951_SM8463.zip › supplementary/Supplementary table1.docx]

Supplementary table 1 for

**MiR-4733-5p promotes gallbladder carcinoma progression via directly targeting Kruppel like factor 7**

Hu et al.

**Table 1.** **The sequences of RNA oligonucleotides used in this study.**

| **RNA oligonucleotides** | **Sequence (5’-3’)** |
| --- | --- |
| miR-4733-5p mimics | AAUCCCAAUGCUAGACCCGGUG |
| miR-4733-5p inhibitor | CACCGGGUCUAGCAUUGGGAUU |
| hsa-miR-551b-3p mimics | GCGACCCAUACUUGGUUUCAG |
| hsa-miR-4443 mimics | UUGGAGGCGUGGGUUUU |
| hsa-miR-4430 mimics | AGGCUGGAGUGAGCGGAG |
| hsa-miR-1185-1-3p mimics | AUAUACAGGGGGAGACUCUUAU |
