# Supplementary material for: MiR-4733-5p promotes gallbladder carcinoma progression via directly targeting kruppel like factor 7
Source: Bioengineered. 2022 Apr 21;13(4):10691–706. doi: 10.1080/21655979.2022.2065951 (PMC9161844; doi:10.1080/21655979.2022.2065951)
Supplement: Supplemental Material [file KBIE_A_2065951_SM8463.zip › supplementary/Supplementary table2.docx]

Supplementary table 2 for

**MiR-4733-5p promotes gallbladder carcinoma progression via directly targeting Kruppel like factor 7**

Hu et al.

**Table 2.** **The sequences of shKLF7 and KLF7 plasmids used in this study**

|  |  | **Sequence (5’-3’)** |
| --- | --- | --- |
| sh-KLF7-1 | sense | GGGUGCCGGAAAGUUUAUATT |
|  | antisense | UAUAAACUUUCCGGCACCCTT |
| sh-KLF7-2 | sense | CAGCAGACAUGCCUUGAAUTT |
|  | antisense | AUUCAAGGCAUGUCUGCUGTT |
| KLF7 | sense | CGCAAATGGGCGGTAGGCGTG |
|  | antisense | TACGGGAAGCAATAGCATGA |
