# Supplementary material for: MiR-4733-5p promotes gallbladder carcinoma progression via directly targeting kruppel like factor 7
Source: Bioengineered. 2022 Apr 21;13(4):10691–706. doi: 10.1080/21655979.2022.2065951 (PMC9161844; doi:10.1080/21655979.2022.2065951)
Supplement: Supplemental Material [file KBIE_A_2065951_SM8463.zip › supplementary/Supplementary table3.docx]

Supplementary table 3 for

**MiR-4733-5p promotes gallbladder carcinoma progression via directly targeting Kruppel like factor 7**

Hu et al.

**Table 3. RT-qPCR primer sequences used in this study.**

| **Primers** | **Forward (****5’-3’)** | **Reverse (5’-3’)** |
| --- | --- | --- |
| miR-4733-5p | CGCGAATCCCAATGCTAGAC | AGTGCAGGGTCCGAGGTATT |
| *U6* | CGATACAGAGAAGATTAGCATGGC | AACGCTTCACGAATTTGCGT |
| *KLF7* | GGTCAAAACCTCACAAACTCTC | CTCTTCTTGTTTTCGGGACATG |
| *ZBTB18* | GTACAGACACATTCGCAAGTTC | TAAGGTCCAGTCTCTGACAGTA |
| *OLR1* | CTGGCATGGAGAAAACTGTTAC | CATCCAAAGACAAGCACTTCTC |
| *CACNG6* | AGAACCACAAAGAAAGAGGTGA | TGAGCACCATGATGATACAGAG |
| *SCD* | CCAAAGACAGCTGAGAACTTCA | TGTTTCTGAAAACTTGTGGTGG |
| *PPIF* | CCAAAGACAGCTGAGAACTTCA | GTGCTTCAGTGTAAAGTTCTCG |
| *RAB30* | AGCTGAAGAATTCTCAGAAGCT | TTGTTCACAAGTGTGTTCTGTC |
| *ACTB* | CTCGCCTTTGCCGATCC | TCTCCATGTCGTCCCAGTTG |
