# Supplementary material for: MiR-4733-5p promotes gallbladder carcinoma progression via directly targeting kruppel like factor 7
Source: Bioengineered. 2022 Apr 21;13(4):10691–706. doi: 10.1080/21655979.2022.2065951 (PMC9161844; doi:10.1080/21655979.2022.2065951)
Supplement: Supplemental Material [file KBIE_A_2065951_SM8463.zip › supplementary/Supplementary table4.docx]

Supplementary table 4 for

**MiR-4733-5p promotes gallbladder carcinoma progression via directly targeting Kruppel like factor 7**

Hu et al.

**Table 4. The five differentially expressed miRNAs in GSE104165 dataset.**

| **miRNA_ID** | **adj.P. Val** | ***t*** | **B** | **logFC** |
| --- | --- | --- | --- | --- |
| hsa-miR-551b-3p | 1.06E-16 | -14.9104 | 34.2811 | -3.5797 |
| hsa-miR-1185-1-3p | 4.35E-12 | 10.2480 | 20.7702 | 2.1466 |
| hsa-miR-4443 | 5.23E-09 | 7.8862 | 12.8973 | 2.4769 |
| hsa-miR-4733-5p | 1.47E-15 | 13.4632 | 30.3894 | 2.4494 |
| hsa-miR-4430 | 1.12E-15 | 13.6333 | 30.8604 | 2.9861 |

adj.P. Val: adjusted P value, FC: fold change.
