# Supplementary figures and images for: MiR-4733-5p promotes gallbladder carcinoma progression via directly targeting kruppel like factor 7
Source: Bioengineered. 2022 Apr 21;13(4):10691–706. doi: 10.1080/21655979.2022.2065951 (PMC9161844; doi:10.1080/21655979.2022.2065951)

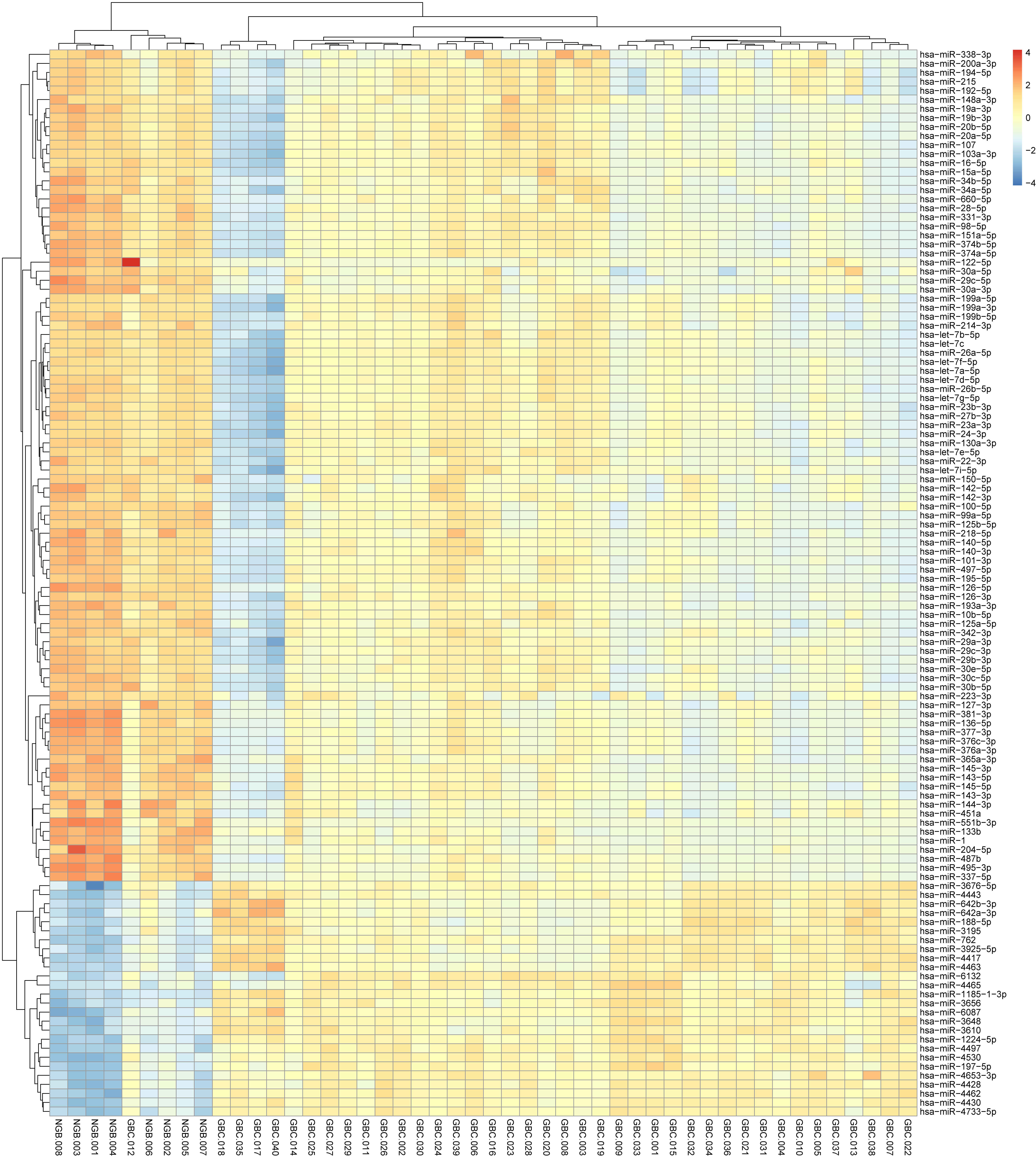

Supplement: Supplemental Material [file KBIE_A_2065951_SM8463.zip › supplementary/Supplementary figure1.tif]

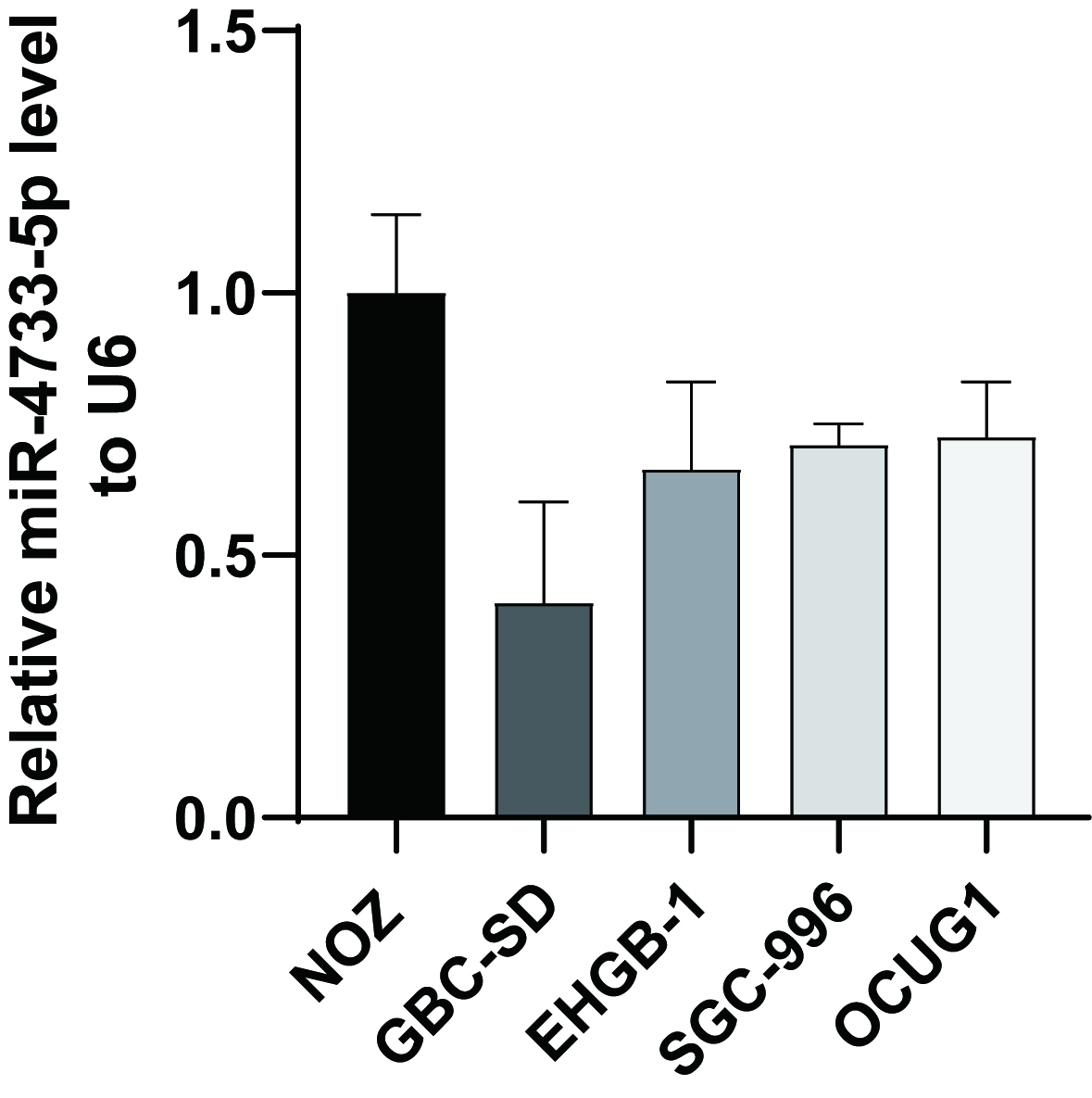

Supplement: Supplemental Material [file KBIE_A_2065951_SM8463.zip › supplementary/Supplementary figure2.tif]
